# Quantifying procedural burden during stent-assisted coil embolization for unruptured intracranial aneurysms: development of the aneurysm endovascular procedural complexity score and internal validation of a multivariable complication model

**DOI:** 10.3389/fneur.2026.1956688

**Published:** 2026-10-30

**Authors:** Yuxiang Huang, E Zhu, Zijun Huang, Yuehang Ouyang, Jian Luo, Liuqing Liu, Xiaosong Huang

**Affiliations:** 1 College of Clinical Medicine, Hunan University of Chinese Medicine, Changsha, China; 2 Department of Neurology, The Second People’s Hospital of Hunan Province (Brain Hospital of Hunan Province), Changsha, China

**Keywords:** internal validation, periprocedural complications, procedural complexity, radiation index, stent-assisted coil embolization, unruptured intracranial aneurysm

## Abstract

Procedural complexity during stent-assisted coil embolization (SAC) is commonly assessed subjectively and is not adequately represented by conventional pre-procedural risk factors. This single-center retrospective cohort study developed the Aneurysm Endovascular Procedural Complexity Score (AEPCS) and internally validated a multivariable complication model incorporating the score in 484 patients with unruptured intracranial aneurysms treated with SAC between January 2018 and December 2025. The AEPCS integrated procedure duration, contrast volume, and the system-reported procedure-level radiation index (mGy) after natural-log transformation, *Z*-standardization, equal-weight averaging, and final standardization. The primary outcome was a composite of symptomatic cerebral infarction, symptomatic intracranial hemorrhage, or all-cause death within 28 days. Major periprocedural complications occurred in 71 patients (14.7%). After adjustment for age, sex, hypertension, diabetes mellitus, posterior-circulation location, aneurysm diameter, neck width, and multiple aneurysms, each 1-standard-deviation increase in the AEPCS was associated with higher odds of a major complication [adjusted odds ratio (OR), 5.25; 95% confidence interval (CI), 3.51–7.86; *p* < 0.001]. The AEPCS showed an area under the receiver operating characteristic curve (AUC) of 0.846 (95% CI, 0.797–0.890); its discrimination was significantly better than that of procedure duration alone but did not differ significantly from the radiation index or contrast volume. In a sensitivity analysis that reincluded 33 patients with definite intraprocedural complications requiring rescue treatment, the association remained materially unchanged (adjusted OR, 5.31; 95% CI, 3.55–7.93; *p* < 0.001), with an AUC of 0.842. The multivariable model had an apparent AUC of 0.853 and an optimism-corrected AUC of 0.834 (95% CI, 0.795–0.890) after 2,000 full-pipeline bootstrap resamples. The AEPCS should therefore be regarded primarily as a standardized composite measure of cumulative procedural burden rather than an outcome-optimized prediction score. External validation is required before clinical implementation.

## 1 Introduction

Despite continued advances in endovascular devices, imaging technology, and perioperative management, major periprocedural complications remain an important concern in the preventive treatment of unruptured intracranial aneurysms (UIAs). Ischemic stroke, intracranial hemorrhage, and procedure-related death may offset the anticipated benefit of aneurysm occlusion and remain important causes of early neurological deterioration following endovascular treatment (1, 2). Stent-assisted coil embolization (SAC) is an established technique for wide-necked and morphologically complex aneurysms because the stent provides a scaffold for coil deployment while preserving the parent artery (3–7). However, the technical demands of SAC vary substantially according to aneurysm morphology, vascular anatomy, catheter accessibility, device manipulation, and intraoperative decision-making. More demanding procedures may require greater procedural resources and may be associated with an increased risk of complications. Nevertheless, procedural complexity is still largely evaluated subjectively.

Existing risk-assessment frameworks for UIAs predominantly focus on characteristics available before treatment. Clinical and anatomical variables have been incorporated into tools for estimating aneurysm rupture, growth, and the appropriateness of preventive intervention (8–10). These approaches support patient selection and pre-procedural decision-making but do not capture the technical burden encountered during the intervention itself. In routine neurointerventional practice, operators readily recognize differences in procedural complexity; however, these judgments are experience-dependent and difficult to reproduce or compare across patients. An objective method for quantifying procedural complexity during SAC is therefore needed.

Procedural complexity is multidimensional and may not be adequately represented by a single intraoperative variable. Procedure duration, contrast volume, and the system-reported procedure-level radiation index (hereafter, radiation index) are routinely recorded procedural metrics. Routine recording of patient radiation exposure is recommended, and radiation indices and fluoroscopy time have been reported in interventional studies (11–13). Together, the three variables may offer a transparent summary of cumulative procedural burden. Procedure duration reflects the overall time and technical effort required to complete the intervention, whereas contrast volume and the radiation index reflect cumulative imaging and fluoroscopic resources used during procedural guidance. These metrics may capture partially overlapping rather than fully independent aspects of procedural burden, particularly because procedure duration and radiation exposure are intrinsically related. Nevertheless, their integration may provide a transparent and standardized summary of the procedural burden encountered during SAC. To our knowledge, no objective composite index based on these routinely collected intraoperative metrics has been developed specifically to quantify procedural complexity during SAC for UIAs.

To address this gap, we developed the Aneurysm Endovascular Procedural Complexity Score (AEPCS) by integrating procedure duration, contrast volume, and the radiation index. We hypothesized that higher AEPCS values would reflect greater cumulative procedural burden and would be associated with higher odds of major periprocedural complications. This study aimed to develop the AEPCS and internally validate a multivariable complication model incorporating it, assess its association with major periprocedural complications following SAC for UIAs, evaluate the robustness of its pre-specified construction, and explore its potential decision utility for early postoperative risk stratification.

## 2 Materials and methods

### 2.1 Study design and population

This single-center retrospective cohort study included consecutive patients with unruptured intracranial aneurysms who underwent stent-assisted coil embolization (SAC) at The Second People’s Hospital of Hunan Province (Brain Hospital of Hunan Province) between January 1, 2018, and December 31, 2025. Reporting of the prediction-model analyses was guided by the Transparent Reporting of a Multivariable Prediction Model for Individual Prognosis or Diagnosis plus Artificial Intelligence (TRIPOD+AI) statement (14). The study protocol was approved by the institutional ethics committee of The Second People’s Hospital of Hunan Province (approval no. 2026K016). The requirement for individual informed consent was waived because of the retrospective nature of the study.

Patients were eligible if they were aged ≥18 years, had at least one unruptured intracranial aneurysm confirmed by digital subtraction angiography, and were intended to undergo SAC during the study period. Patients were excluded from the primary analytic cohort if they had a ruptured intracranial aneurysm; ultimately underwent simple coiling, balloon-assisted coiling, flow diversion, parent artery occlusion, or another endovascular treatment without stent assistance; underwent treatment for an arteriovenous malformation, dural arteriovenous fistula, or another cerebrovascular lesion during the same procedure; experienced a definite intraprocedural complication requiring rescue treatment that could directly affect the AEPCS components; or had incomplete data required for AEPCS construction, covariate adjustment, or primary-outcome assessment. The exclusion rule for recognized intraprocedural complications requiring rescue treatment was determined before the primary AEPCS–outcome statistical analysis.

A total of 556 potentially eligible patients were initially screened. Of these, 23 patients were excluded because the intended SAC strategy was converted intraoperatively to flow-diverter treatment. Thirty-three patients were excluded from the primary analytic cohort because they experienced a definite intraprocedural complication requiring rescue treatment, including cerebral vasospasm in 15 patients, thrombus formation in 11 patients, and aneurysm rupture with hemorrhage in 7 patients. These exclusions were applied during cohort assembly before the primary AEPCS–outcome analysis because the complication itself and subsequent rescue treatment could directly prolong procedure duration or increase contrast administration and radiation exposure, thereby introducing temporal ambiguity and potential reverse causation. An additional 16 patients were excluded because of incomplete information required for analysis: 3 had missing previous-medical-history data, 5 had missing contrast-volume data, and 8 lacked 28-day outcome data. The final primary analytic cohort therefore comprised 484 patients. No procedure was abandoned after initiation, and no patient experienced technical failure without definitive endovascular treatment.

For sensitivity analysis addressing potential reverse causation and selection resulting from exclusion of recognized intraprocedural complications, the 33 patients with definite intraprocedural complications were reincluded, resulting in an expanded cohort of 517 patients. Patients converted to flow-diverter treatment and patients with incomplete data remained excluded.

For patients with multiple intracranial aneurysms, the aneurysm treated with SAC during the index procedure was considered the index aneurysm. When more than one aneurysm was treated during the same procedure, the aneurysm that primarily determined the treatment strategy and procedural complexity was designated as the index aneurysm.

### 2.2 Endovascular procedure

All procedures were performed under general anesthesia by the same neurointerventional team using the same single-plane digital subtraction angiography system (GE Optima; GE HealthCare, Chicago, IL, United States). The selection of the stent, coils, microcatheters, and procedural strategy was determined by the treating neurointerventionalists according to aneurysm morphology, parent-artery anatomy, vascular accessibility, and intraoperative technical requirements.

All patients received dual antiplatelet therapy with aspirin and clopidogrel for at least 3 days before the procedure. Systemic heparinization was administered during the procedure according to the institutional protocol. A microcatheter was advanced into the aneurysm sac, and a self-expanding intracranial stent was deployed across the aneurysm neck to provide scaffold support for coil embolization. The sequence of stent deployment and coil placement was selected according to the aneurysm configuration and operator judgment. Additional stents or coils were used when required to achieve satisfactory aneurysm occlusion while preserving parent-artery patency.

After the procedure, dual antiplatelet therapy was continued for 6 months, followed by aspirin monotherapy for an additional 6 months, unless adjustment was clinically indicated.

### 2.3 Data collection

Demographic and clinical variables included age, sex, hypertension, diabetes mellitus, previous stroke, cardiac disease, hyperlipidemia, smoking history, and alcohol consumption. Aneurysm-related variables included aneurysm location, anterior or posterior circulation location, morphology, maximum diameter, neck width, and the presence of multiple aneurysms.

Procedural variables included the number of stents and coils used, procedure duration, contrast volume, radiation index, immediate angiographic result, and intraprocedural complications. Follow-up variables included angiographic recurrence, retreatment, follow-up duration, and clinical outcomes.

Procedure duration was defined as the interval from successful arterial puncture to removal of the arterial sheath, as documented in the procedural record, and was expressed in minutes. Contrast volume was defined as the total volume of iodinated contrast medium administered during the procedure and was expressed in milliliters. The radiation variable was the radiation index recorded by the angiography system in milligray (mGy). The specific dosimetric quantity was not separately identified in the analytic dataset. All procedures were performed using the same angiography system (Supplementary Table S2).

Two investigators independently reviewed the electronic medical records, procedural reports, imaging findings, and other available source records using a standardized data-collection form. Classification of definite intraprocedural complications additionally incorporated intraoperative angiographic findings, anesthesia records, and documentation of rescue treatment. Disagreements were resolved through discussion, with adjudication by a senior neurointerventionalist when consensus could not be reached. When classifying intraprocedural complications and determining cohort exclusions, both investigators and the senior adjudicator were blinded to final AEPCS values and 28-day outcome status.

### 2.4 Development of the AEPCS

The Aneurysm Endovascular Procedural Complexity Score (AEPCS) was developed to quantify cumulative procedural burden using three routinely recorded intraoperative metrics: procedure duration, contrast volume, and radiation index.

Because the distributions of these variables were right-skewed, each variable was transformed using the natural logarithm:


$$X_{\mathrm{ij}}^{*}=\ln(X_{\mathrm{ij}})$$

where $X_{\mathrm{ij}}$ represents the original value of procedural metric j for patient i , and j represents procedure duration, contrast volume, or radiation index.

Each log-transformed variable was then standardized using the 484-patient development-cohort mean and sample standard deviation:


$$Z_{\mathrm{ij}}=\frac{X_{\mathrm{ij}}^{*}-\bar{X_{j}^{*}}}{\mathrm{SD}(X_{j}^{*})}$$

$$Z_{\text{duration},i}=\frac{\ln(D_{i})-\bar{\ln(D)}}{\mathrm{SD}[\ln(D)]}$$

$$Z_{\text{contrast},i}=\frac{\ln(C_{i})-\bar{\ln(C)}}{\mathrm{SD}[\ln(C)]}$$

$$Z_{\text{radiation},i}=\frac{\ln(R_{i})-\bar{\ln(R)}}{\mathrm{SD}[\ln(R)]}$$

where $D_{i}$ , $C_{i}$ , and $R_{i}$ represent the procedure duration, contrast volume, and radiation index, respectively, for patient i .

The three standardized metrics were assigned equal weights and averaged to generate a preliminary composite score:


$$\text{AEPC}S_{i}^{\mathrm{raw}}=\frac{Z_{\text{duration},i}+Z_{\text{contrast},i}+Z_{\text{radiation},i}}{3}$$

The preliminary composite score was subsequently standardized to obtain the final AEPCS:


$$\text{AEPC}S_{i}=\frac{\text{AEPC}S_{i}^{\mathrm{raw}}-\bar{\text{AEPC}S^{\mathrm{raw}}}}{\mathrm{SD}(\text{AEPC}S^{\mathrm{raw}})}$$

$$\text{AEPC}S_{i}=Z[\frac{Z(\ln D_{i})+Z(\ln C_{i})+Z(\ln R_{i})}{3}]$$

Accordingly, the final AEPCS had a development-cohort mean of 0 and a standard deviation of 1. Higher AEPCS values indicated greater cumulative procedural burden. Equal weighting was specified *a priori* to maintain interpretability and to avoid deriving outcome-dependent weights that could increase the risk of overfitting.

To enable independent calculation and external validation, the centering and scaling parameters derived from the development cohort were fixed and are reported in Supplementary Table S1. These parameters should be retained unchanged when applying the AEPCS to an external patient or validation cohort. Based on the development-cohort parameters, the AEPCS can be calculated directly using the following rounded coefficients:


$$\begin{array}{l} \text{AEPCS}=-25.707665+1.169453\ln(D)\\ +1.665535\ln(C)+1.458399\ln(R) \end{array}$$

where D , C , and R represent procedure duration in minutes, contrast volume in milliliters, and radiation index in milligray, respectively (Supplementary Table S3).

### 2.5 Outcome definitions

The primary outcome was a major periprocedural complication occurring within 28 days after SAC. The composite outcome comprised symptomatic cerebral infarction, symptomatic intracranial hemorrhage, or all-cause death within 28 days after the procedure.

Symptomatic cerebral infarction was defined as a newly developed focal neurological deficit relative to pre-procedural neurological status accompanied by a corresponding acute ischemic lesion on computed tomography or magnetic resonance imaging. Symptomatic intracranial hemorrhage was defined as a newly detected intracranial hemorrhage on postoperative imaging accompanied by neurological deterioration relative to pre-procedural status. Death was defined as death from any cause within 28 days after the procedure. Mortality within this window was confirmed using recorded death dates and source medical records. Patients who experienced more than one component event were counted only once in the composite primary outcome.

For cohort assembly, a definite intraprocedural complication was defined as an angiographically or clinically recognized adverse event occurring during the procedure that required rescue treatment. These events included cerebral vasospasm, thrombus formation, and aneurysm rupture with hemorrhage. Because such events and their treatment could themselves alter procedure duration, contrast administration, and radiation exposure, these patients were excluded from the primary analytic cohort to reduce temporal ambiguity and potential reverse causation.

Patients without an intraprocedurally recognized complication requiring rescue treatment were not excluded solely because a clinical complication became evident after the procedure. Provided that the other eligibility criteria were met, they remained in the primary cohort and were counted as outcome cases if they fulfilled the original 28-day endpoint criteria.

In the expanded sensitivity cohort, the original definition of the 28-day composite primary outcome was retained. An intraprocedural adverse event was therefore not automatically classified as a primary-outcome event unless the patient subsequently met the pre-specified criteria for symptomatic cerebral infarction, symptomatic intracranial hemorrhage, or all-cause death.

### 2.6 Statistical analysis

Continuous variables were assessed for distributional normality using graphical methods and the Shapiro–Wilk test. Normally distributed variables are presented as mean and standard deviation, whereas non-normally distributed variables are presented as median and interquartile range. Categorical variables are presented as frequencies and percentages.

Baseline and procedural characteristics were compared between patients with and without major periprocedural complications. The independent-samples *t* test or Mann–Whitney U test was used for continuous variables, as appropriate. The Pearson chi-square test or Fisher exact test was used for categorical variables.

Correlations among procedure duration, contrast volume, radiation index, and the AEPCS were assessed using Spearman rank correlation coefficients. The distributions of the three procedural metrics and the AEPCS were examined graphically.

The association between the AEPCS and major periprocedural complications was evaluated using binary logistic regression. The AEPCS was analyzed primarily as a continuous variable, with the odds ratio representing the change in the odds of a major complication associated with each 1-unit increase in the score, corresponding to 1 standard deviation in the development cohort. An unadjusted model and a multivariable-adjusted model were constructed.

The adjusted model included age, sex, hypertension, diabetes mellitus, posterior-circulation aneurysm location, maximum aneurysm diameter, neck width, and multiple aneurysms. These covariates were selected *a priori* according to clinical relevance and previously reported associations with outcomes after endovascular aneurysm treatment rather than through univariable statistical screening. Adjusted associations are reported as odds ratios with 95% confidence intervals. Multicollinearity was assessed using variance inflation factors.

Discrimination of the AEPCS score alone and of each individual procedural metric was quantified using empirical receiver operating characteristic analysis. Percentile 95% confidence intervals for AUCs and paired differences in AUC were estimated using 2,000 stratified bootstrap resamples, with patients resampled separately within outcome groups. This score-level analysis was distinguished from internal validation of the multivariable prediction model.

Calibration was assessed for the multivariable logistic regression model incorporating AEPCS and the eight clinical and aneurysm-related covariates. Apparent and optimism-corrected calibration curves were estimated using LOWESS smoothing with a span of two-thirds and no robustifying iterations. Calibration intercept and slope were estimated jointly by logistic regression of the observed outcome on the model linear predictor; the Brier score summarized squared prediction error.

Potential decision utility was explored using decision-curve analysis by comparing the pre-specified clinical model with and without the AEPCS across threshold probabilities of 5–50% (15). This threshold range was examined descriptively to characterize potential decision utility rather than to define a validated clinical intervention threshold. The analysis used apparent predictions from the development cohort and was not corrected for optimism. The decision context was hypothetical intensified postoperative neurological surveillance; the default strategies represented offering this additional surveillance to all patients or to none.

Because random division of the cohort into development and validation subsets would reduce statistical efficiency and yield unstable estimates, the full primary cohort was used for score development and model estimation. Internal validation was performed using 2,000 ordinary patient-level bootstrap resamples with replacement (random seed 20,261,004) (16). Within each bootstrap sample, the complete score-construction and model-fitting procedure was repeated, including natural-log transformation of the three procedural metrics, estimation of centering and scaling parameters, reconstruction and final standardization of the AEPCS, and fitting of the multivariable logistic regression model. Model performance was evaluated in both the bootstrap sample and the original cohort to estimate optimism. When predicting the original cohort, the centering and scaling parameters estimated within that bootstrap resample were retained; the original cohort was not independently restandardized. Mean optimism across the 2,000 resamples was subtracted from the apparent performance to obtain optimism-corrected estimates of the AUC, Brier score, calibration intercept, and calibration slope. Calibration-curve optimism was estimated on a fixed prediction grid by comparing the smoothed bootstrap-training and original-cohort calibration curves. Resamples lacking prediction support at a grid point were omitted at that point without extrapolation. All 2,000 model fits were successful.

Ninety-five percent confidence intervals for optimism-corrected model performance were estimated using a two-stage patient-level bootstrap procedure (17). We generated 2,000 ordinary outer bootstrap samples from the 484-patient development cohort. Within each outer sample, the complete score-construction and model-fitting pipeline was repeated, and optimism was estimated using 2,000 ordinary inner bootstrap samples. All score parameters and model coefficients were re-estimated within each inner training sample and retained when evaluating predictions in the corresponding outer sample. Each outer-sample corrected estimate equaled its apparent performance minus the mean inner-sample training-minus-test difference. Corrected confidence limits were obtained from the 2.5th and 97.5th percentiles of the outer corrected estimates using R type-6 quantiles. Apparent AUC and Brier confidence limits were obtained from their outer-sample apparent distributions. Apparent joint calibration intercepts and slopes were treated as structural fitted-model values of 0 and 1, respectively, and their confidence intervals were omitted. The CI analysis used random seed 20,261,006. Resamples with separation were retained using the original unpenalized logistic fitting settings; fitting diagnostics and a convergence-tolerance sensitivity analysis are reported in Supplementary Table S9.

Several additional sensitivity and robustness analyses were performed. First, to assess the potential influence of excluding definite intraprocedural complications, the primary association analysis was repeated in the expanded cohort of 517 patients after reinclusion of the 33 patients with recognized intraprocedural complications requiring rescue treatment. AEPCS values for these patients were calculated using the fixed centering and scaling parameters derived from the 484-patient development cohort.

Second, to assess component redundancy and the robustness of the pre-specified equal-weight formulation, three leave-one-component-out variants were constructed by separately omitting procedure duration, contrast volume, or radiation index. The remaining log-transformed standardized components were averaged with equal weights and the resulting composite was standardized before association and discrimination analyses. Pairwise differences in AUC between the original AEPCS and alternative specifications were evaluated using paired stratified bootstrap resampling.

Third, principal component analysis (PCA) of the three log-transformed and standardized procedural metrics was performed as an exploratory assessment of their underlying dimensional structure. The proportion of variance explained by the first principal component, component loadings, correlation with the pre-specified AEPCS, and its association and discrimination for the primary outcome were examined.

Exploratory restricted cubic spline analysis assessed the functional form of the AEPCS–outcome association, with adjustment for the same eight covariates. A three-knot specification at the 10th, 50th, and 90th percentiles was used to limit model complexity. Likelihood-ratio tests compared the spline model with the covariate-only model for the overall association and with the linear-score model for nonlinearity, using two and one degrees of freedom, respectively. Sensitivity to knot specification was examined using four knots at the 5th, 35th, 65th, and 95th percentiles.

No formal sample-size calculation was performed because all eligible consecutive patients treated during the predefined study period were considered for inclusion. The primary linear-score multivariable model contained nine predictor parameters and 71 primary outcome events.

The final primary analytic dataset contained no missing values. Among the 556 initially screened patients, 16 (2.9%) were excluded because of incomplete information required for analysis: 3 had missing previous-medical-history information, 5 had missing contrast-volume measurements required for AEPCS construction, and 8 lacked 28-day outcome data. Because the overall amount of missing data was small and involved heterogeneous variables, no statistical imputation was performed. Complete-case analysis was used, and the potential for selection bias associated with exclusion of incomplete cases was considered when interpreting the findings.

All statistical tests were two-sided, and a *p* value <0.05 was considered statistically significant. Statistical analyses were performed using R software, version 4.4.3 (R Foundation for Statistical Computing, Vienna, Austria). The complete model coefficients, intercept, variable coding, and prediction equation are reported in Supplementary Table S7.

## 3 Results

### 3.1 Study population and clinical characteristics

A total of 556 potentially eligible patients were screened. Twenty-three patients were excluded because the intended SAC strategy was converted intraoperatively to flow-diverter treatment, 33 were excluded from the primary analytic cohort because of definite intraprocedural complications requiring rescue treatment (15 cerebral vasospasm, 11 thrombus formation, and 7 aneurysm rupture with hemorrhage), and 16 were excluded because of incomplete data (3 with missing previous-medical-history data, 5 with missing contrast-volume data, and 8 with unavailable 28-day outcome data). The final primary analytic cohort comprised 484 patients (Supplementary Figure S1). No procedure was abandoned after initiation or ended in technical failure without definitive endovascular treatment.

The median age was 61 years [interquartile range (IQR), 54–70 years], 157 patients (32.4%) were male, and 311 (64.3%) had hypertension. Posterior-circulation aneurysms were present in 170 patients (35.1%), and 160 patients (33.1%) had multiple aneurysms.

Major periprocedural complications occurred in 71 patients (14.7%). Among these patients, 55 experienced symptomatic cerebral infarction and 16 experienced symptomatic intracranial hemorrhage. Twenty patients (4.1% of the primary cohort) died within 28 days after the procedure, including 14 after symptomatic cerebral infarction and 6 after symptomatic intracranial hemorrhage.

Compared with patients without major complications, those with major complications more frequently had hypertension (74.6% vs. 62.5%, *p* = 0.048) and multiple aneurysms (43.7% vs. 31.2%, *p* = 0.040). They also required a greater number of coils [median, 8 (IQR, 6–11) vs. 7 (IQR, 5–10); *p* = 0.047]. No significant between-group differences were observed in age, sex, diabetes mellitus, previous stroke, cardiac disease, hyperlipidemia, smoking history, alcohol consumption, aneurysm location, aneurysm morphology, maximum aneurysm diameter, neck width, number of stents, or immediate Raymond–Roy classification. Detailed clinical, aneurysm, and procedural characteristics are presented in Table 1.

**Table 1:** Clinical, aneurysm, and procedural characteristics according to major periprocedural complications.

Characteristic	Overall (*N* = 484)	No major periprocedural complications (*N* = 413)	Major periprocedural complications (*N* = 71)	*p* value
Clinical characteristics				
Age, years	61 (54–70)	61 (54–69)	64 (56–72)	0.245
Male sex, *n* (%)	157 (32.4)	138 (33.4)	19 (26.8)	0.269
Hypertension, *n* (%)	311 (64.3)	258 (62.5)	53 (74.6)	0.048
Diabetes mellitus, *n* (%)	58 (12.0)	45 (10.9)	13 (18.3)	0.076
Previous stroke, *n* (%)	117 (24.2)	98 (23.7)	19 (26.8)	0.582
Cardiac disease, *n* (%)	85 (17.6)	76 (18.4)	9 (12.7)	0.241
Hyperlipidemia, *n* (%)	129 (26.7)	110 (26.6)	19 (26.8)	0.982
Smoking history, *n* (%)	68 (14.0)	55 (13.3)	13 (18.3)	0.263
Alcohol consumption, *n* (%)	40 (8.3)	30 (7.3)	10 (14.1)	0.054
Aneurysm characteristics				
Posterior circulation location, *n* (%)	170 (35.1)	141 (34.1)	29 (40.8)	0.274
Aneurysm morphology, *n* (%)				0.352
Saccular	306 (63.2)	264 (63.9)	42 (59.2)	
Fusiform	9 (1.9)	9 (2.2)	0 (0.0)	
Dissecting	15 (3.1)	14 (3.4)	1 (1.4)	
Irregular	154 (31.8)	126 (30.5)	28 (39.4)	
Maximum aneurysm diameter, mm	6.2 (4.8–8.2)	6.2 (4.7–8.0)	6.5 (5.1–9.3)	0.056
Neck width, mm	4.0 (3.1–5.1)	4.0 (3.1–5.0)	4.5 (3.3–5.5)	0.065
Multiple aneurysms, *n* (%)	160 (33.1)	129 (31.2)	31 (43.7)	0.040
Procedural characteristics				
Number of stents	1 (1–1)	1 (1–1)	1 (1–1)	0.186
Number of coils	7 (5–10)	7 (5–10)	8 (6–11)	0.047
Immediate Raymond-Roy class, *n* (%)				0.483
Class I	317 (65.5)	274 (66.3)	43 (60.6)	
Class II	161 (33.3)	134 (32.4)	27 (38.0)	
Class III	6 (1.2)	5 (1.2)	1 (1.4)	
Contrast volume, mL	194 (172–236)	187 (169–223)	255 (219–286)	<0.001
Procedure duration, min	170 (140–210)	160 (130–200)	220 (185–270)	<0.001
Radiation index, mGy	1,647 (1,455–2,049)	1,603 (1,411–1,905)	2,389 (1,847–2,675)	<0.001
AEPCS	−0.06 (−0.68–0.71)	−0.20 (−0.77–0.40)	1.24 (0.34–1.61)	<0.001

Data are presented as median (interquartile range) or number (percentage), as appropriate. Comparisons between patients with and without major periprocedural complications were performed using the Mann–Whitney *U* test for continuous variables and the Pearson chi-square test or Fisher exact test for categorical variables, as appropriate. Major periprocedural complications were defined as symptomatic cerebral infarction, symptomatic intracranial hemorrhage, or all-cause death within 28 days after the procedure. Radiation index denotes the system-reported procedure-level index recorded in mGy; the specific dosimetric quantity was not separately identified in the analytic dataset. *p* values are two-sided, and *p* < 0.05 was considered statistically significant.

### 3.2 Distribution and interrelationships of procedural metrics

The overall median procedure duration was 170 min (IQR, 140–210 min), the median contrast volume was 194 mL (IQR, 172–236 mL), and the median radiation index was 1,647 mGy (IQR, 1,455–2,049 mGy). All three procedural metrics showed right-skewed distributions. The AEPCS had a median of −0.06 (IQR, −0.68 to 0.71), with a mean of 0 and a standard deviation of 1 by construction (Figure 1).

**Figure 1:**
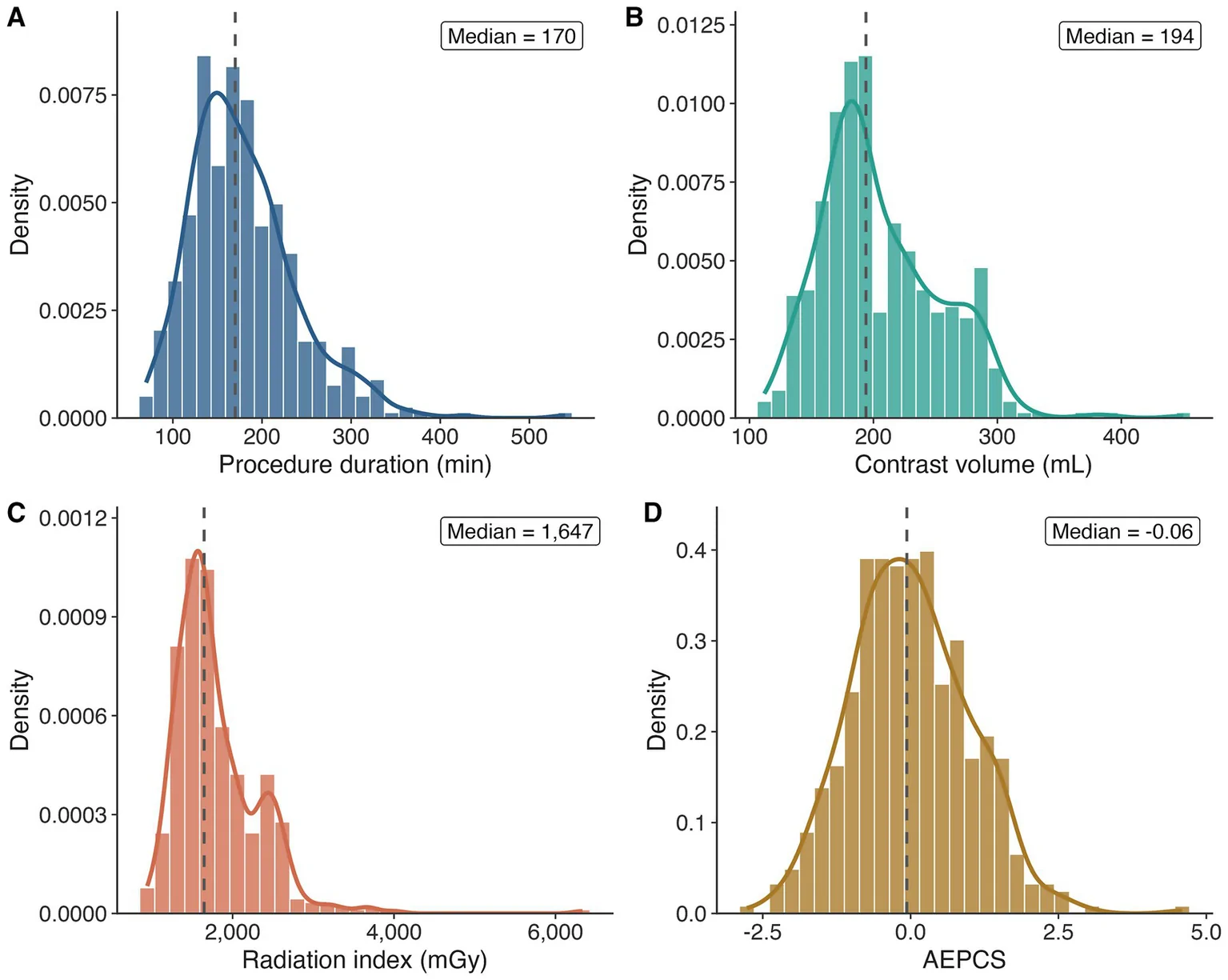
Distributions of the procedural metrics and the Aneurysm Endovascular Procedural Complexity Score. Histograms with overlaid kernel-density curves show the distributions of **(A)** procedure duration, **(B)** contrast volume, **(C)** radiation index, and **(D)** the Aneurysm Endovascular Procedural Complexity Score (AEPCS) among 484 patients undergoing stent-assisted coil embolization. The dashed vertical line in each panel indicates the median value. The median procedure duration, contrast volume, radiation index, and AEPCS were 170 min, 194 mL, 1,647 mGy, and −0.06, respectively.

Procedure duration, contrast volume, and radiation index were positively correlated with one another. The correlation was particularly strong between procedure duration and radiation index (Spearman *ρ* = 0.948, *p* < 0.001), whereas procedure duration was moderately correlated with contrast volume (*ρ* = 0.500, *p* < 0.001), and contrast volume was moderately correlated with radiation index (*ρ* = 0.527, *p* < 0.001). The AEPCS was strongly correlated with procedure duration (*ρ* = 0.925, *p* < 0.001) and radiation index (*ρ* = 0.933, *p* < 0.001) and was also positively correlated with contrast volume (*ρ* = 0.764, *p* < 0.001) (Figure 2).

**Figure 2:**
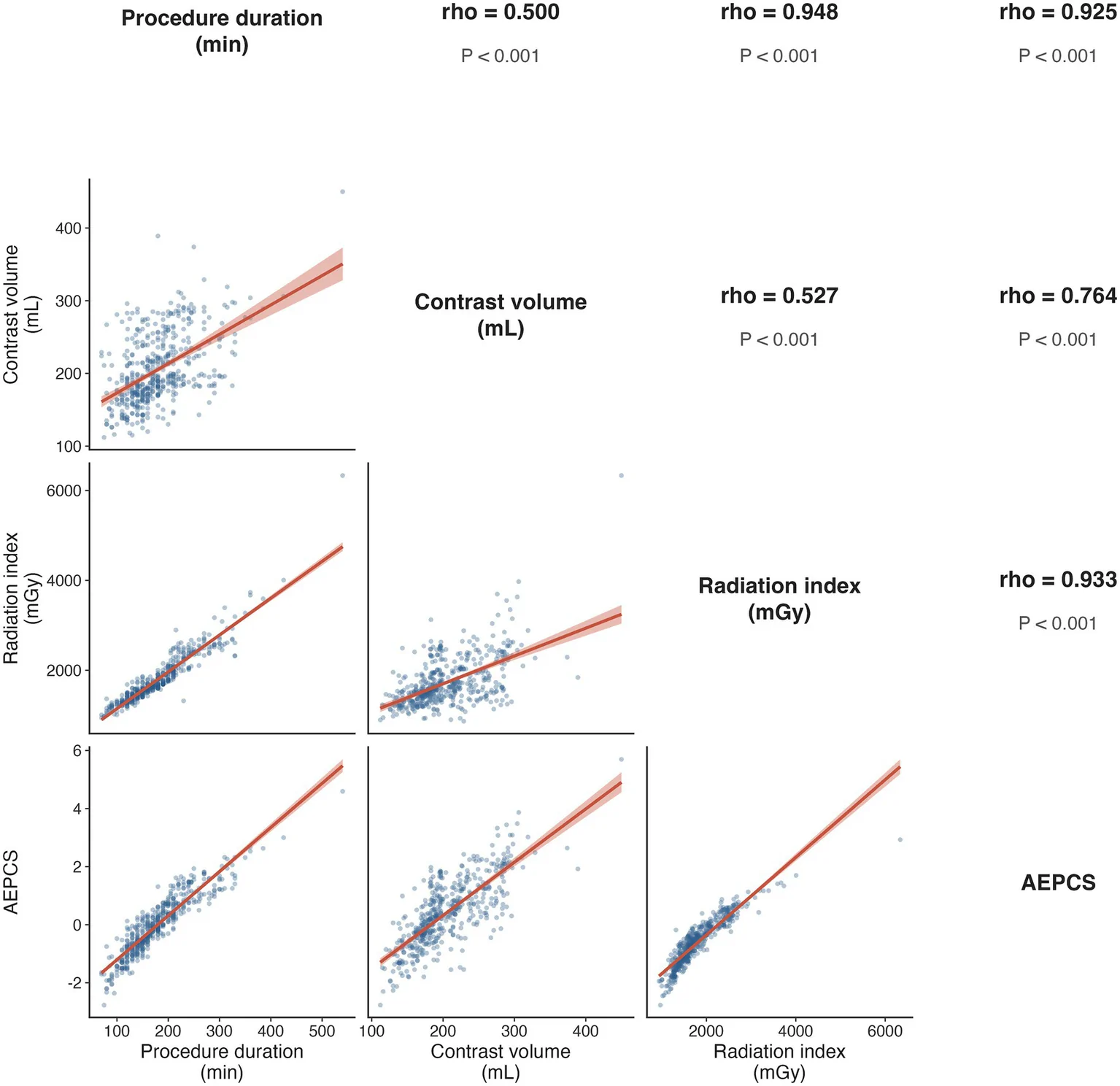
Pairwise correlations among procedure duration, contrast volume, radiation index, and the Aneurysm Endovascular Procedural Complexity Score. The lower panels show pairwise scatterplots with fitted trend lines, and the upper panels report Spearman rank correlation coefficients and corresponding *p* values. All pairwise correlations were statistically significant (*p* < 0.001).

Patients who developed major periprocedural complications had substantially greater procedural exposure than those without complications. Median procedure duration was 220 min (IQR, 185–270 min) in the complication group compared with 160 min (IQR, 130–200 min) in the non-complication group (*p* < 0.001). Corresponding median contrast volumes were 255 mL (IQR, 219–286 mL) and 187 mL (IQR, 169–223 mL), respectively (*p* < 0.001), while median radiation index values were 2,389 mGy (IQR, 1,847–2,675 mGy) and 1,603 mGy (IQR, 1,411–1,905 mGy), respectively (*p* < 0.001). The median AEPCS was also significantly higher in patients with major complications than in those without complications [1.24 (IQR, 0.34–1.61) vs. −0.20 (IQR, −0.77 to 0.40); *p* < 0.001].

### 3.3 Association between the AEPCS and major periprocedural complications

In unadjusted logistic regression, each 1-standard-deviation increase in the AEPCS was associated with increased odds of a major periprocedural complication [odds ratio (OR), 5.28; 95% confidence interval (CI), 3.58–7.78; *p* < 0.001].

After adjustment for age, sex, hypertension, diabetes mellitus, posterior-circulation aneurysm location, maximum aneurysm diameter, neck width, and multiple aneurysms, the association remained essentially unchanged (adjusted OR, 5.25; 95% CI, 3.51–7.86; *p* < 0.001) (Table 2).

**Table 2:** Associations of the AEPCS and pre-specified covariates with major periprocedural complications.

Variable	Unadjusted OR (95% CI)	*p* value	Adjusted OR (95% CI)	*P* value
AEPCS, per 1-SD increase	5.28 (3.58–7.78)	<0.001	5.25 (3.51–7.86)	<0.001
Age, per year	1.00 (0.98–1.03)	0.805	0.99 (0.96–1.02)	0.493
Male sex	0.73 (0.41–1.28)	0.270	0.77 (0.40–1.49)	0.437
Hypertension	1.77 (1.00–3.13)	0.050	1.49 (0.73–3.04)	0.268
Diabetes mellitus	1.83 (0.93–3.61)	0.079	2.03 (0.87–4.71)	0.100
Posterior circulation location	1.33 (0.80–2.23)	0.275	1.25 (0.68–2.30)	0.476
Maximum aneurysm diameter, per mm	1.06 (1.00–1.12)	0.053	1.02 (0.93–1.13)	0.664
Neck width, per mm	1.04 (0.92–1.17)	0.540	1.01 (0.85–1.21)	0.910
Multiple aneurysms	1.71 (1.02–2.85)	0.041	0.94 (0.50–1.75)	0.841

Odds ratios and 95% confidence intervals were estimated using binary logistic regression. Unadjusted ORs were estimated using separate univariable logistic regression models. The adjusted ORs were estimated from a single multivariable model including the AEPCS, age, sex, hypertension, diabetes mellitus, posterior-circulation location, maximum aneurysm diameter, neck width, and multiple aneurysms. The odds ratio for the AEPCS represents the change in the odds of a major periprocedural complication associated with each 1-unit increase in the score, equivalent to 1 standard deviation in the development cohort. Odds ratios for age, maximum aneurysm diameter, and neck width represent changes per 1-year, 1-mm, and 1-mm increase, respectively. Reference categories were female sex, absence of hypertension or diabetes mellitus, anterior-circulation location, and a single aneurysm. *p* values are two-sided, and *p* < 0.05 was considered statistically significant.

None of the pre-specified clinical or aneurysm-related covariates reached statistical significance after inclusion of the AEPCS in the adjusted model. Variance inflation factors ranged from 1.02 to 1.54, indicating no material multicollinearity among the variables included in the model.

### 3.4 Discriminative performance of the AEPCS

The AEPCS demonstrated good discrimination for major periprocedural complications, with an empirical area under the receiver operating characteristic curve (AUC) of 0.846 (95% CI, 0.797–0.890). Radiation index had an AUC of 0.835 (95% CI, 0.783–0.884), contrast volume had an AUC of 0.829 (95% CI, 0.783–0.873), and procedure duration had an AUC of 0.776 (95% CI, 0.717–0.831) (Figure 3).

**Figure 3:**
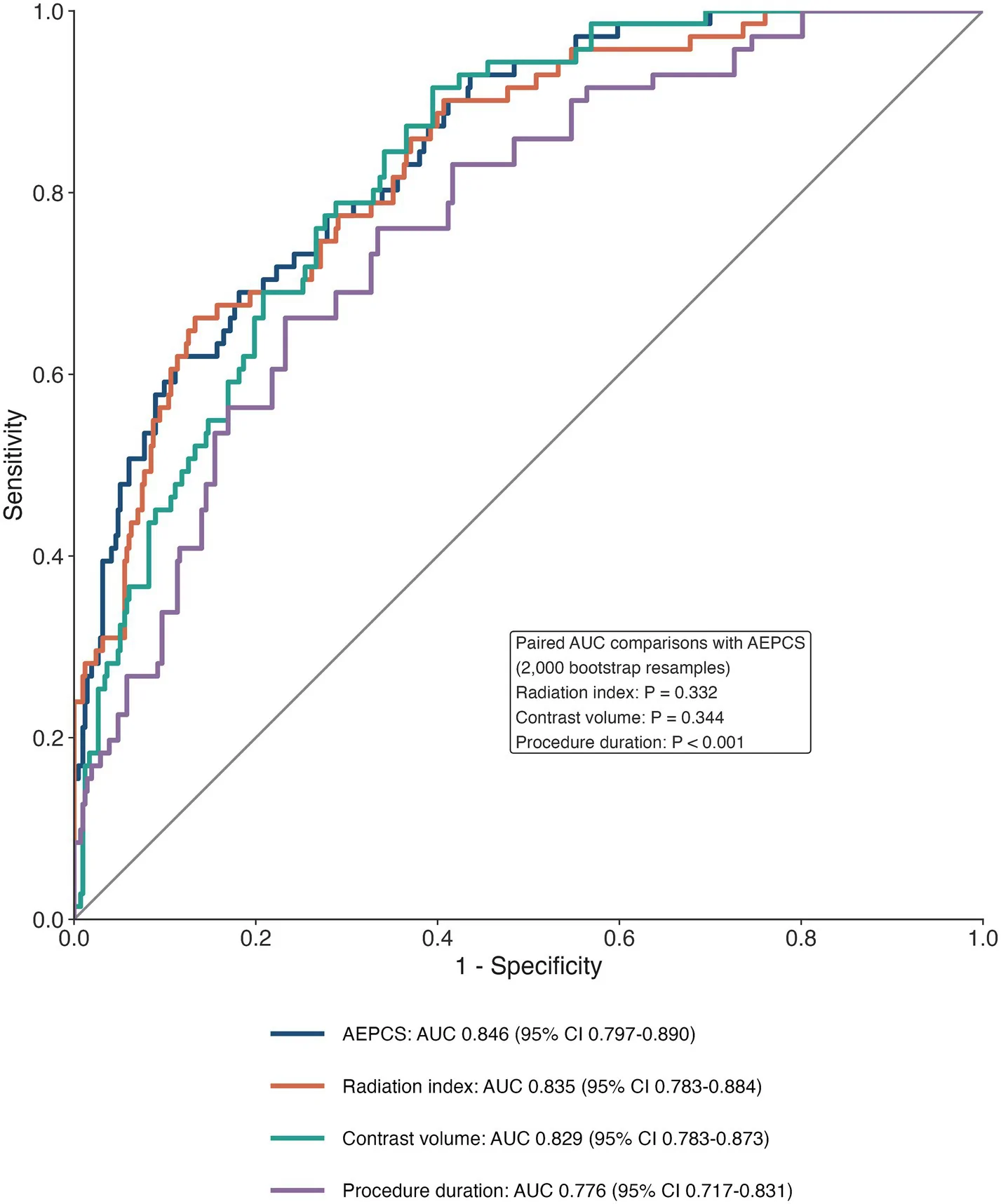
Receiver operating characteristic curves for the Aneurysm Endovascular Procedural Complexity Score and its individual components. Receiver operating characteristic curves compare the discrimination of the AEPCS, radiation index, contrast volume, and procedure duration for major periprocedural complications. Areas under the curve were calculated empirically, and percentile 95% confidence intervals were estimated using 2,000 stratified bootstrap resamples. Paired bootstrap comparisons showed that the AEPCS significantly outperformed procedure duration (*p* < 0.001), whereas its discrimination did not differ significantly from that of radiation index (*p* = 0.332) or contrast volume (*p* = 0.344).

In paired comparisons based on 2,000 stratified bootstrap resamples, the AUC of the AEPCS was not significantly different from that of radiation index (difference in AUC, 0.010; 95% CI, −0.011 to 0.034; *p* = 0.332) or contrast volume (difference in AUC, 0.017; 95% CI, −0.018 to 0.053; *p* = 0.344). The AEPCS showed significantly better discrimination than procedure duration alone (difference in AUC, 0.070; 95% CI, 0.052–0.091; *p* < 0.001). Thus, the AEPCS did not demonstrate superior discrimination over all individual procedural metrics (Supplementary Table S4).

### 3.5 Internal validation and calibration

The multivariable model incorporating the AEPCS and the pre-specified clinical and aneurysm-related covariates had an apparent AUC of 0.853 (95% CI, 0.819–0.903). After full-pipeline internal validation with 2,000 bootstrap resamples, the optimism-corrected AUC was 0.834 (95% CI, 0.795–0.890).

The apparent Brier score was 0.089 (95% CI, 0.069–0.103), and the optimism-corrected Brier score was 0.096 (95% CI, 0.075–0.110). The apparent joint calibration intercept and slope were 0.000 and 1.000, respectively. The optimism-corrected joint calibration intercept was −0.115 (95% CI, −0.178 to −0.086), and the optimism-corrected calibration slope was 0.897 (95% CI, 0.870–0.913). The point estimates suggested optimism in apparent performance, while the confidence intervals quantified uncertainty in internally validated performance (Figure 4 and Supplementary Table S9).

**Figure 4:**
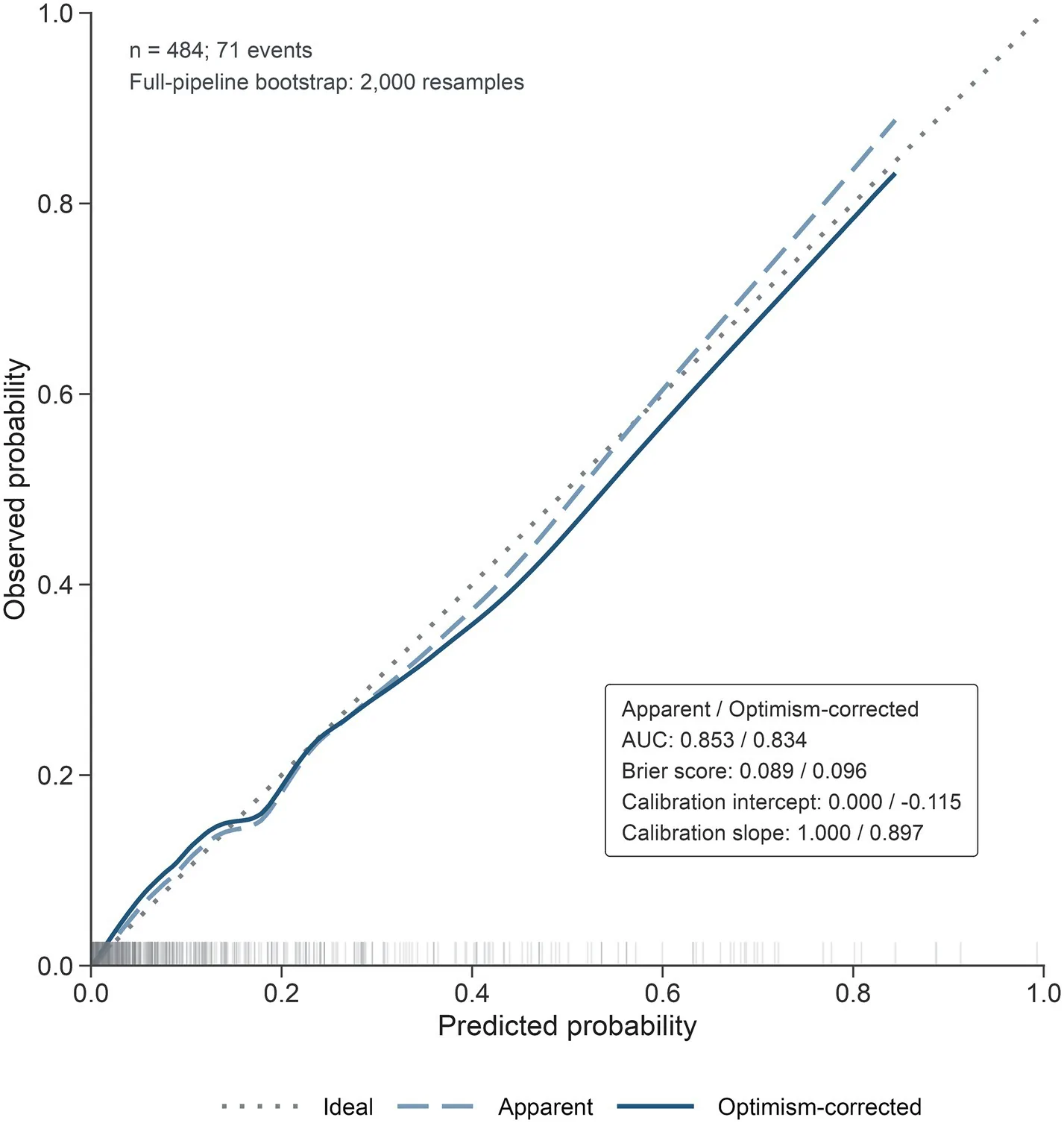
Calibration of the multivariable model for major periprocedural complications. The calibration plot compares predicted and observed probabilities for the multivariable model incorporating the AEPCS, age, sex, hypertension, diabetes mellitus, posterior-circulation aneurysm location, maximum aneurysm diameter, neck width, and multiple aneurysms. Full-pipeline internal validation was performed using 2,000 bootstrap resamples. The apparent and optimism-corrected Brier scores were 0.089 and 0.096, respectively; the corresponding joint calibration intercepts were 0.000 and −0.115, and the calibration slopes were 1.000 and 0.897. The diagonal reference line represents perfect calibration. Score-construction parameters and model coefficients were re-estimated within every resample, and bootstrap-derived score parameters were retained for predictions in the original cohort. The curves use LOWESS smoothing; rug marks show the predicted-probability distribution. Two-stage bootstrap 95% confidence intervals for the model-performance measures are reported in Supplementary Table S9. These numerical intervals describe uncertainty in the performance measures and are not confidence bands for the plotted calibration curves.

### 3.6 Exploratory decision-curve analysis

Exploratory decision-curve analysis compared the pre-specified clinical model with a combined model incorporating the AEPCS. The clinical model included age, sex, hypertension, diabetes mellitus, posterior-circulation location, maximum aneurysm diameter, neck width, and multiple aneurysms. This analysis used apparent development-cohort predictions and was not corrected for optimism. Across threshold probabilities from 5 to 50%, the combined clinical model plus AEPCS showed greater net benefit than the clinical model alone and the default strategies of classifying all patients or no patients as high risk (Figure 5). These apparent differences in net benefit are exploratory; they do not establish a validated intervention threshold or demonstrate improved clinical outcomes from AEPCS-guided surveillance.

**Figure 5:**
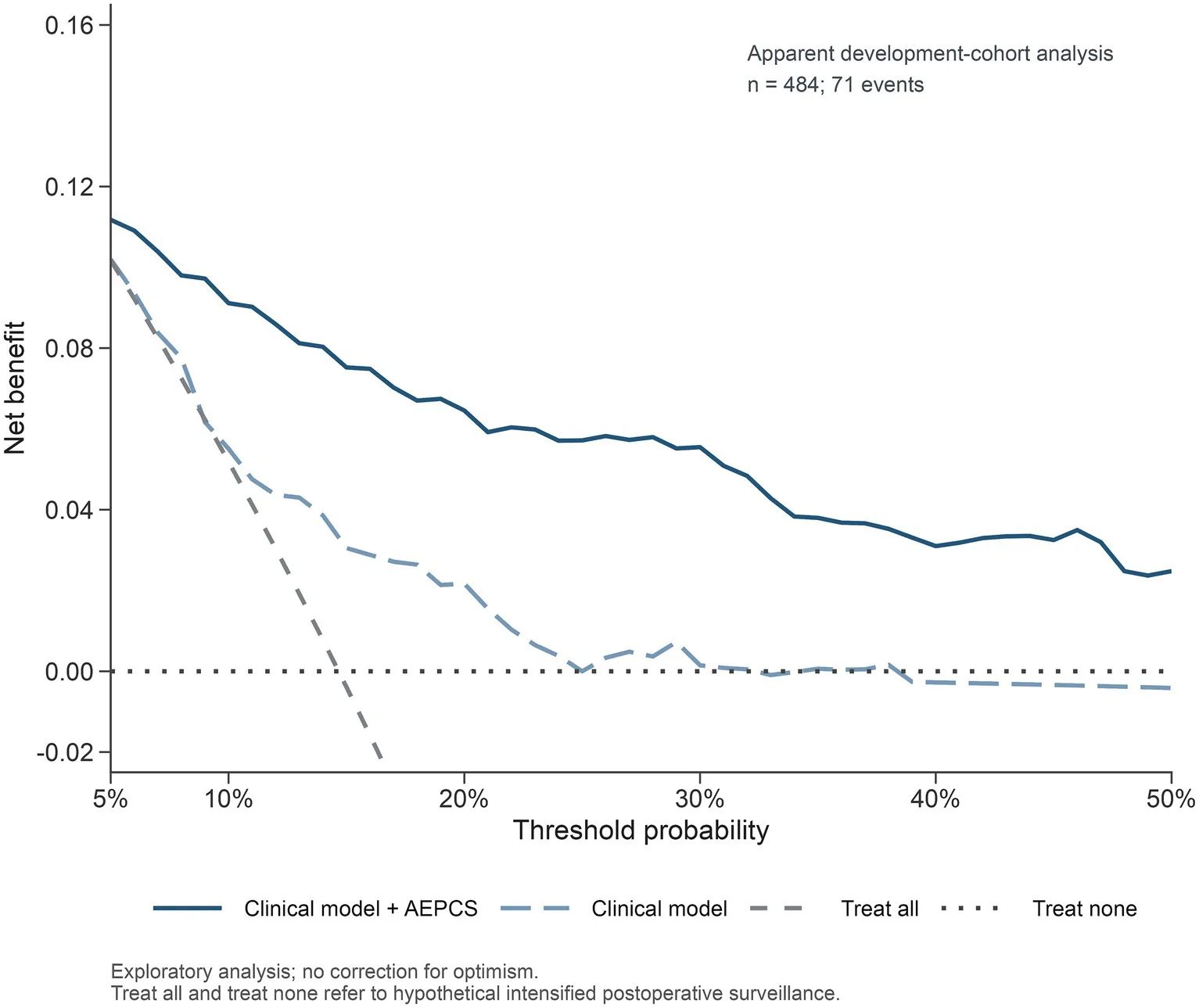
Decision-curve analysis of the clinical model with and without the Aneurysm Endovascular Procedural Complexity Score. Net benefit was compared across threshold probabilities of 5–50% for the clinical model alone, the clinical model incorporating the AEPCS, and the hypothetical strategies of intensified postoperative surveillance for all patients or for none. The clinical model included age, sex, hypertension, diabetes mellitus, posterior-circulation aneurysm location, maximum aneurysm diameter, neck width, and multiple aneurysms. The model incorporating the AEPCS showed greater net benefit than the clinical model alone across the evaluated threshold-probability range; this analysis was exploratory and was not intended to establish a validated intervention threshold. The curves show apparent development-cohort performance and were not corrected for optimism.

### 3.7 Sensitivity analysis addressing intraprocedural complications

To evaluate the potential influence of excluding definite intraprocedural complications, the analysis was repeated after reincluding the 33 patients who had experienced an intraprocedural complication requiring rescue treatment, resulting in an expanded cohort of 517 patients. Of the 33 reincluded patients, 2 met the original primary endpoint, both because of symptomatic cerebral infarction; intraprocedural adverse events that did not meet the pre-specified primary-outcome criteria were not counted as outcome events. Overall, 73 of 517 patients (14.1%) met the primary outcome in the expanded cohort.

The association between AEPCS and major periprocedural complications remained materially unchanged. Each 1-unit increase in AEPCS, equivalent to 1 standard deviation in the 484-patient development cohort, was associated with an unadjusted OR of 5.40 (95% CI, 3.66–7.95; *p* < 0.001) and an adjusted OR of 5.31 (95% CI, 3.55–7.93; *p* < 0.001). The AUC of the AEPCS was 0.842 (95% CI, 0.795–0.883), compared with 0.846 in the primary cohort (Supplementary Table S5). These findings indicate that reinclusion of patients with recognized intraprocedural complications did not materially alter the magnitude of the association or discrimination of the AEPCS.

### 3.8 Robustness of AEPCS construction and functional form

Leave-one-component-out analyses showed that the association between composite procedural burden and the primary outcome remained statistically significant regardless of which component was omitted. The adjusted ORs per 1-standard-deviation increase were 6.04 (95% CI, 3.96–9.20) for the variant excluding procedure duration, 3.94 (95% CI, 2.76–5.64) for the variant excluding contrast volume, and 4.82 (95% CI, 3.25–7.15) for the variant excluding radiation index (all *p* < 0.001). The corresponding AUCs were 0.864, 0.809, and 0.836, respectively (Supplementary Table S6).

Notably, the *post hoc* two-component variant excluding procedure duration showed modestly higher discrimination than the pre-specified three-component AEPCS (difference in AUC, 0.018; 95% CI, 0.008–0.030; *p* = 0.002).

In exploratory PCA, the first principal component explained 78.0% of the total variance, with positive loadings of 0.615 for procedure duration, 0.480 for contrast volume, and 0.625 for radiation index. The first principal component was almost perfectly correlated with the pre-specified equal-weight AEPCS (*r* = 0.998) and demonstrated a similar association with the primary outcome (adjusted OR, 5.08; 95% CI, 3.41–7.56; *p* < 0.001) and similar discrimination (AUC, 0.842).

In the three-knot restricted cubic spline model, the overall association was significant (likelihood-ratio *p* < 0.001), while the test for departure from linearity on the log-odds scale was not significant (P for nonlinearity = 0.802). In the exploratory four-knot sensitivity model, the nonlinearity test yielded *p* = 0.047 (Supplementary Figure S2 and Supplementary Table S8). These results indicate sensitivity to knot specification and do not establish that the underlying relationship is necessarily linear.

## 4 Discussion

In this single-center cohort of 484 patients undergoing SAC for UIAs, AEPCS provided a standardized summary of cumulative procedural burden and was strongly associated with major periprocedural complications. The association persisted in the expanded 517-patient cohort and across alternative component specifications. Its discrimination was comparable to that of radiation index and contrast volume individually, and the *post hoc* variant excluding procedure duration had a modestly higher AUC. Internal validation characterized the performance of the multivariable complication model incorporating AEPCS; it did not independently validate the score as a measure of technical difficulty.

The AEPCS was designed to quantify a dimension of aneurysm treatment that is widely recognized by neurointerventionalists but is not adequately represented in existing risk-assessment frameworks. Available tools, such as the SAFETEA scores, estimate the risk of complications after preventive aneurysm treatment primarily from patient, aneurysm, and treatment characteristics known before intervention (18). Such models are valuable for treatment selection and informed consent but cannot capture unanticipated technical challenges that arise during the procedure. Difficult vascular access, unstable microcatheter positioning, repeated device adjustment, incomplete stent opening, coil instability, and additional angiographic acquisitions may substantially increase procedural burden despite apparently similar preprocedural anatomy. The AEPCS therefore should not be regarded as a replacement for preprocedural risk assessment, but rather as a complementary postprocedural measure describing the cumulative burden recorded during completed SAC.

Major periprocedural complications occurred in 14.7% of the present cohort. Direct comparison with previous studies should be cautious because complication rates vary according to rupture status, treatment technique, antiplatelet protocol, imaging surveillance, follow-up window, and outcome definition. A systematic review of stent-assisted aneurysm treatment of ruptured aneurysms demonstrated that both thromboembolic and hemorrhagic events are clinically relevant procedural hazards, while a large elective UIA series using stent-assisted coiling or flow diversion reported thromboembolic and hemorrhagic complication rates of 5.75 and 4.75%, respectively (19, 20). Postprocedural diffusion-weighted imaging studies further indicate that ischemic lesions may occur more frequently than clinically apparent neurological events (21). In addition, ischemic and hemorrhagic risks following intracranial stenting are influenced by antiplatelet selection, individual platelet response, intraprocedural anticoagulation, and device-related thrombogenicity (22). These multiple mechanisms support interpreting the AEPCS as a marker of cumulative procedural burden rather than as a direct biological cause of complications.

Procedure duration is an intuitive measure of procedural burden. A prolonged procedure may indicate difficult catheterization, repeated microcatheter reshaping, unstable access, multiple stent or coil deployments, device exchange, or the need to resolve unexpected technical problems. In a consecutive cohort of patients undergoing coil embolization, Lee et al. found that longer procedure time was associated with thromboembolic events and suggested that procedural duration may represent an important potentially modifiable procedural factor (23). Nevertheless, procedure duration may also be influenced by vascular access, sheath management, anticoagulation-related considerations, and workflow delays that do not directly represent intracranial technical complexity. Consistent with this limitation, procedure duration had the lowest individual discrimination among the three AEPCS components in the present cohort.

Contrast volume captures a related but distinct dimension of procedural burden. Repeated angiographic runs, updated roadmaps, working-angle adjustments, and verification of parent-vessel or branch patency may increase contrast administration even when the total procedure time is not markedly prolonged. Greater contrast exposure may also have clinical relevance in selected patients. A study of coil embolization for UIAs found that longer procedure duration and greater contrast dose were associated with postprocedural contrast leakage, while another cohort identified higher contrast dosage as a risk factor for contrast-induced encephalopathy after endovascular aneurysm treatment (24, 25). However, the primary outcome in the present study consisted predominantly of symptomatic infarction, intracranial hemorrhage, and death rather than isolated contrast neurotoxicity. Contrast volume should therefore be interpreted mainly as an objective procedural surrogate, although direct contrast-related neurological effects cannot be entirely excluded.

The system-reported radiation index was used as a recorded indicator of the imaging-related procedural burden generated during catheter navigation, fluoroscopic guidance, angiographic acquisition, stent deployment, and coil placement. Previous comparative studies have shown that neuroendovascular treatment strategies differ substantially in procedure time, fluoroscopy time, and radiation exposure, with SAC generally requiring greater imaging resources than simpler coiling strategies (26). Forbrig et al. reported longer fluoroscopy times for combined endovascular techniques and lower dose–area product with a low-dose DSA protocol. Dose–area product was associated with aneurysm size and patient age, although differences between endovascular techniques were not statistically significant (27). Radiation exposure itself is unlikely to cause acute ischemic or hemorrhagic complications within the 28-day outcome window. Within the AEPCS, the recorded radiation index serves as an indicator of imaging-related procedural burden.

Procedure duration and the radiation index were highly correlated (Spearman *ρ* = 0.948), indicating substantial overlap. Equal weighting was selected *a priori* to provide an interpretable index without estimating outcome-dependent component weights. The leave-one-component-out associations and the dominant PCA component, which explained 78.0% of variance and correlated strongly with AEPCS, describe the shared structure of the included metrics. They do not provide independent validation against operator-rated difficulty or another external standard of technical complexity. The three-component formulation and its weights should therefore be regarded as a pragmatic index specification whose transportability requires further evaluation.

At the same time, the two-component variant excluding procedure duration demonstrated modestly but statistically significantly higher discrimination than the pre-specified AEPCS. This finding suggests that procedure duration contributes partly redundant predictive information, particularly given its strong correlation with the radiation index. However, this alternative specification was evaluated *post hoc* after examination of the present data. Replacing the pre-specified AEPCS solely because the simplified score produced a higher AUC in the development cohort would constitute outcome-driven model optimization and could exaggerate apparent performance. We therefore retained the original three-component AEPCS as the primary index and regard the two-component formulation as hypothesis-generating. Future external validation should determine whether a simplified formulation provides better transportability or predictive performance.

The adjusted association between AEPCS and major periprocedural complications was substantial, with an adjusted OR of 5.25 per 1-standard-deviation increase. This estimate describes an increase in odds rather than a fivefold increase in absolute risk. The association changed minimally after adjustment for age, sex, hypertension, diabetes mellitus, posterior-circulation location, aneurysm diameter, neck width, and multiplicity. The three-knot spline model did not detect departure from linearity on the log-odds scale, whereas the four-knot sensitivity model yielded a different nonlinearity result. The reported OR is therefore a summary under the primary linear-score model; the functional form remains uncertain and warrants evaluation in larger independent cohorts.

None of the individual baseline clinical or aneurysm-related covariates remained statistically significant after inclusion of the AEPCS. This should not be interpreted as evidence that these factors are clinically unimportant. The AEPCS is a downstream procedural measure and may partly integrate the cumulative effects of anatomical difficulty, vascular access, device manipulation, and unanticipated technical challenges related to baseline characteristics. In addition, with 71 primary outcome events and nine predictor parameters, statistical precision for detecting modest independent associations of individual baseline covariates was limited.

The ROC findings require particularly cautious interpretation. The AEPCS achieved good discrimination and significantly outperformed procedure duration alone, but its AUC was not statistically superior to those of the radiation index or contrast volume individually. Furthermore, the *post hoc* score excluding procedure duration achieved slightly higher discrimination than the original AEPCS. The principal contribution of the AEPCS is therefore not a universal improvement in predictive accuracy over every individual component, but the transparent integration and standardization of routinely recorded procedural exposures into a single summary measure. AUC alone is also insufficient to determine the usefulness of a clinical model; calibration, internal validation, and potential decision consequences should be considered together when evaluating model performance (28).

Full-pipeline internal validation produced an optimism-corrected AUC of 0.834 (95% CI, 0.795–0.890) compared with an apparent AUC of 0.853. The optimism-corrected Brier score was 0.096, the corrected calibration slope was 0.897, and the corrected joint calibration intercept was −0.115. The corresponding confidence intervals are reported in Supplementary Table S9. The reduction in AUC and the calibration slope below 1 suggested overfitting in the apparent model, consistent with the limited number of outcome events relative to the number of model parameters. The confidence intervals provide estimates of uncertainty in internally validated performance. Bootstrap correction estimates optimism within the development cohort but does not establish external validity or determine whether calibration will be maintained across different patient populations, procedural teams, or angiography systems.

The exploratory decision curves were based on apparent development-cohort predictions and may overestimate decision utility. AEPCS is available only after completion of SAC, so its potential use lies in postoperative assessment rather than treatment selection. Intensified neurological surveillance is a possible decision context for future investigation, but no AEPCS cutoff or predicted-risk threshold has been validated. The present decision curves do not demonstrate that AEPCS-guided surveillance prevents complications or improves outcomes; an evolving complication may also have contributed to the recorded procedural burden before the score became available.

This study has several strengths. The cohort was restricted to UIAs treated exclusively with completed SAC, reducing heterogeneity associated with rupture status and fundamentally different endovascular techniques. All procedures were performed under general anesthesia by the same neurointerventional team using the same angiography system, improving the internal consistency of measurements of procedure duration and the radiation index. The AEPCS was based entirely on routinely recorded objective variables and did not require additional imaging segmentation or subjective operator grading. Covariates were selected *a priori* rather than through univariable statistical screening. In addition, full-pipeline bootstrap resampling was used rather than randomly dividing a moderately sized dataset into inefficient development and validation subsets, and multiple sensitivity analyses were performed to assess temporality, component redundancy, weighting assumptions, and the functional form of the AEPCS–outcome association.

Several limitations should be acknowledged. Both analytic cohorts were restricted to completed SAC procedures; therefore, the findings may not generalize to patients requiring intraoperative conversion to flow diversion or to those undergoing other endovascular treatment strategies. The retrospective single-center design limits generalizability and creates potential selection and information biases. Team experience, equipment, imaging protocols, and antiplatelet practices were institution-specific and may have changed during 2018–2025. Complete-case analysis excluded 16 of 556 screened patients (2.9%) because of missing medical-history information, contrast measurements, or outcomes; missingness related to patient characteristics or procedural difficulty cannot be excluded. Residual confounding by baseline neurological status and unmeasured technical characteristics is also possible.

Temporal ambiguity remains important. Exclusion of 33 patients with recognized intraprocedural complications requiring rescue treatment may have preferentially removed more difficult procedures. Although reinclusion yielded similar association estimates, it cannot eliminate reverse causation: an ischemic or thromboembolic process may have begun during the procedure before clinical or angiographic recognition. The composite outcome also combined infarction, hemorrhage, and death, which differ in mechanisms and severity, and there were insufficient events for reliable component-specific modeling.

The measurements capture procedural resource use and may not correspond directly to technical difficulty. Procedure duration includes vascular access, sheath management, and workflow time, while the system-reported radiation index may depend on equipment configuration, patient size, projection geometry, and acquisition settings (29, 30). Equivalent radiation quantities must be confirmed before applying fixed score parameters across systems. No independent operator-rating standard was available, and the strong component correlations and PCA findings do not establish construct validity. Original pre-consensus abstraction ratings were not retained, preventing calculation of quantitative inter-rater agreement.

The primary model included 71 events for nine predictor parameters and showed optimism after bootstrap correction. The *post hoc* superiority of the variant excluding procedure duration and the sensitivity of the spline nonlinearity test to knot specification indicate uncertainty about the score formulation and functional form. Neither external validation nor prospective evaluation of an AEPCS-guided management strategy was performed.

## 5 Conclusion

AEPCS integrates procedure duration, contrast volume, and the system-reported radiation index into a standardized measure of cumulative procedural burden during SAC for UIAs. Higher values were associated with major periprocedural complications in the primary and expanded cohorts. Discrimination was not significantly greater than that of the radiation index or contrast volume alone, and a *post hoc* two-component variant performed modestly better. Multicenter external validation, comparison with independent measures of technical difficulty, and prospective evaluation of postoperative decision use are required before clinical implementation.

## Data Availability

The datasets presented in this article are not readily available because the datasets generated and/or analyzed during the current study are not publicly available due to patient privacy and ethical restrictions. The de-identified data may be available from the authors upon reasonable request and approval from the institutional ethics committee. Requests to access the datasets should be directed to YH, nideexplorer@163.com.
