# Supplementary material for: Quantifying procedural burden during stent-assisted coil embolization for unruptured intracranial aneurysms: development of the aneurysm endovascular procedural complexity score and internal validation of a multivariable complication model
Source: Front Neurol. 2026 Oct 30;17:1956688. doi: 10.3389/fneur.2026.1956688 (PMC13759386; doi:10.3389/fneur.2026.1956688)
Supplement: Supplementary file 1 [file Table_1.docx]

Supplementary Material

# Supplementary Tables

Supplementary Table 1. Development-cohort parameters required for calculation of the AEPCS

| Procedural metric | Unit | Transformation | Development-cohort mean | Development-cohort SD |
| --- | --- | --- | --- | --- |
| Procedure duration | min | Natural logarithm | 5.137385 | 0.324235 |
| Contrast volume | mL | Natural logarithm | 5.297672 | 0.227661 |
| Radiation index | mGy | Natural logarithm | 7.457683 | 0.259996 |
| Raw composite score | — | Equal-weight average of the three standardized metrics | 0.000000 | 0.879095 |

Note: All centering and scaling parameters were derived from the development cohort of 484 patients. When calculating the AEPCS in an external patient or validation cohort, these development-cohort parameters should be retained unchanged rather than re-estimated from the external dataset. Procedure duration is expressed in minutes, contrast volume in milliliters, and the radiation index in milligray. Natural logarithms are used for all three procedural metrics.

Supplementary Table 2. Definitions and coding of demographic, clinical, aneurysm-related, procedural, and outcome variables

| Variable category | Variable | Definition / Coding |
| --- | --- | --- |
| Demographic characteristics | Age | Age at admission, recorded in years |
|  | Sex | Male = 1; Female = 0 |
| Medical history | Hypertension | History of hypertension before admission: Yes = 1; No = 0 |
|  | Diabetes mellitus | History of diabetes mellitus: Yes = 1; No = 0 |
|  | Previous stroke | History of stroke before the index SAC procedure: Yes = 1; No = 0 |
|  | Hyperlipidemia | History of hyperlipidemia: Yes = 1; No = 0 |
|  | Cardiovascular disease | History of ischemic heart disease or other diagnosed cardiac disease: Yes = 1; No = 0 |
|  | Smoking history | Current or previous smoking: Yes = 1; No = 0 |
|  | Alcohol consumption | Current or previous alcohol consumption: Yes = 1; No = 0 |
| Aneurysm characteristics | Rupture status | Ruptured aneurysm = 1; Unruptured aneurysm = 0 |
|  | Location | Anterior circulation = 0; Posterior circulation = 1 |
|  | Morphology | Saccular = 1; Fusiform = 2; Dissecting = 3; Irregular = 4 |
|  | Maximum diameter | Maximum aneurysm diameter measured on preoperative imaging (mm) |
|  | Neck width | Maximum aneurysm neck width measured on preoperative imaging (mm) |
|  | Multiple aneurysms | Presence of multiple intracranial aneurysms: Yes = 1; No = 0 |
| Procedural characteristics | Number of stents | Total number of implanted intracranial stents |
|  | Number of coils | Total number of implanted detachable coils |
|  | Procedure duration | Time from arterial puncture to removal of the arterial sheath (minutes) |
|  | Contrast medium volume | Total iodinated contrast agent administered during procedure (mL) |
|  | System-reported radiation index | Procedure-level radiation index recorded by the angiography system (mGy) |
| Outcome variables | Major periprocedural complications | Symptomatic cerebral infarction, symptomatic intracranial hemorrhage, or all-cause death occurring within 28 days after SAC: Yes = 1; No = 0. Patients with more than one component event were counted once. |
|  | Symptomatic cerebral infarction | Newly developed focal neurological deficit relative to preprocedural status, accompanied by a corresponding acute ischemic lesion on CT or MRI, within 28 days after SAC. |
|  | Symptomatic intracranial hemorrhage | Newly detected intracranial hemorrhage on postoperative imaging, accompanied by neurological deterioration relative to preprocedural status, within 28 days after SAC. |
|  | All-cause Death | All-cause mortality within 28 days after treatment |

Note: Definitions apply to the primary and expanded analytic cohorts. Numeric codes for descriptive categories do not imply inclusion as ordinal predictors. The adjusted association model included AEPCS and eight prespecified baseline covariates: age, sex, hypertension, diabetes mellitus, posterior-circulation location, maximum aneurysm diameter, neck width, and multiple aneurysms. Posterior-circulation location was coded as 1 and anterior-circulation location as 0 in regression analyses. Model coefficients and predictor coding are provided in Supplementary Table 7. The primary outcome comprised symptomatic cerebral infarction, symptomatic intracranial hemorrhage, or all-cause death within 28 days after SAC; patients experiencing more than one component event were counted only once.

Supplementary Table 3. Components, standardization procedure, and calculation of the Aneurysm Endovascular Procedural Complexity Score

| Step | Calculation |
| --- | --- |
| 1. Log transformation | Take the natural logarithm of procedure duration (*D*, minutes), contrast volume (*C*, milliliters), and the radiation index (*R*, milligray). |
| 2. Component standardization | Standardize each log-transformed component using its mean and sample standard deviation estimated in the 484-patient development cohort. |
| 3. Equal-weight averaging | Calculate the arithmetic mean of the three standardized components. |
| 4. Final standardization | Standardize this average using its development-cohort mean and sample standard deviation to obtain the AEPCS. |
| 5. Application to other patients | Retain all development-cohort centering and scaling parameters when calculating AEPCS in the expanded cohort or an external cohort. |

Note: The AEPCS is calculated as follows:

$$Z_{D}=\frac{ln\left( D \right)-\mu_{D}}{S_{D}}$$

$$Z_{C}=\frac{ln\left( C \right)-\mu_{C}}{S_{C}}$$

$$Z_{R}=\frac{ln\left( R \right)-\mu_{R}}{S_{R}}$$

$$A=\frac{Z_{D}+Z_{C}+Z_{R}}{3}$$

$$AEPCS=\frac{A-\mu_{A}}{S_{A}}$$

Here, $\mu_{D}$, $\mu_{C}$, $\mu_{R}$ and $S_{D}$, $S_{C}$, $S_{R}$ are the development-cohort means and sample standard deviations of the log-transformed components. The mean and sample standard deviation of the equal-weight average were ($\mu_{A}$=0.000000000) and ($S_{A}$=0.879094896), respectively. All required parameters are provided in Supplementary Table 1.

Higher AEPCS values indicate greater recorded procedural burden. One AEPCS unit equals one standard deviation in the development cohort. No median-based high/low categorization was used in the primary analyses. Validation cohorts should not be independently restandardized. During full-pipeline bootstrap internal validation, all centering and scaling parameters were re-estimated within each resample and retained when predicting the original cohort. Equivalence of the radiation quantity should be confirmed before applying the fixed parameters across equipment platforms.

Supplementary Table 4. Discrimination of AEPCS compared with individual procedural metrics

| Reference metric | Metric AUC  (95% CI) | AEPCS AUC  (95% CI) | Δ AUC, AEPCS minus metric  (95% CI) | Paired bootstrap P |
| --- | --- | --- | --- | --- |
| Procedure duration | 0.776 (0.717-0.831) | 0.846 (0.797-0.890) | 0.070 (0.052-0.091) | <0.001 |
| Contrast volume | 0.829 (0.783-0.873) | 0.846 (0.797-0.890) | 0.017 (-0.018-0.053) | 0.344 |
| System-reported radiation index | 0.835 (0.783-0.884) | 0.846 (0.797-0.890) | 0.010 (-0.011-0.034) | 0.332 |

Note: Analyses included all 484 patients in the primary cohort. AUCs represent empirical discrimination of AEPCS alone and each individual procedural metric. Percentile confidence intervals and paired comparisons used 2,000 stratified patient-level bootstrap resamples, with outcome strata preserved and the same sampled patients used for each paired comparison. ΔAUC was calculated as AUC of AEPCS minus AUC of the reference metric. P values were two-sided.
These are apparent score-level discrimination comparisons, distinct from the optimism-corrected performance of the multivariable model incorporating AEPCS and eight baseline covariates.
AEPCS, Aneurysm Endovascular Procedural Complexity Score; AUC, area under the receiver operating characteristic curve; CI, confidence interval.

Supplementary Table 5. Primary and expanded cohort analyses

| Measure | Primary cohort | Expanded cohort |
| --- | --- | --- |
| Patients | 484 | 517 |
| Composite outcome | 71 (14.7%) | 73 (14.1%) |
| Unadjusted OR (95% CI), per score unit | 5.28 (3.58–7.78) | 5.40 (3.66–7.95) |
| Adjusted OR (95% CI), per score unit | 5.25 (3.51–7.86) | 5.31 (3.55–7.93) |
| AUC (95% CI) | 0.846 (0.797–0.890) | 0.842 (0.795–0.883) |

Note: One score unit equals one SD in the development cohort. The expanded cohort retained the development-cohort score parameters. Of the 33 reincluded rescue-treatment cases, two met the original primary endpoint, both through symptomatic infarction. Intraprocedural adverse events were not automatically counted as primary outcomes. All association P values were <0.001. AUC percentile CIs used 2,000 stratified bootstrap resamples. Adjusted models included age, sex, hypertension, diabetes mellitus, posterior-circulation location, maximum aneurysm diameter, neck width, and multiple aneurysms.

Supplementary Table 6. Component and PCA specifications

| Specification | Adjusted OR (95% CI) | AUC (95% CI) | ΔAUC versus original (95% CI) | Paired bootstrap P |
| --- | --- | --- | --- | --- |
| AEPCS | 5.25 (3.51–7.86) | 0.846 (0.797–0.890) | Reference | — |
| Without duration | 6.04 (3.96–9.20) | 0.864 (0.820–0.904) | 0.018 (0.008–0.030) | 0.002 |
| Without contrast | 3.94 (2.76–5.64) | 0.809 (0.754–0.862) | −0.037 (−0.054–−0.021) | 0.001 |
| Without radiation | 4.82 (3.25–7.15) | 0.836 (0.786–0.882) | −0.010 (−0.022–0.003) | 0.133 |
| PCA | 5.08 (3.41–7.56) | 0.842 (0.793–0.887) | −0.004 (−0.008–−0.001) | 0.016 |

Note: Variants were standardized in the primary cohort; ORs represent one variant-specific development SD and adjust for the same eight covariates. Association P values were <0.001. ΔAUC is variant minus original AEPCS. Paired stratified bootstrap used 2,000 resamples. PCA was exploratory: PC1 explained 78.0% of variance, with positive-oriented loadings of 0.615, 0.480, and 0.625 for duration, contrast, and the radiation index, respectively, and correlation r=0.998 with AEPCS. These alternative specifications were not independently validated.

Supplementary Table 7. Complete prediction model coefficients

| Term | Combined model β | Clinical model β |
| --- | --- | --- |
| Intercept | -2.518564570 | -2.170664402 |
| AEPCS | 1.658514659 | Not included |
| Age | -0.009346300 | -0.008031494 |
| Male sex | -0.262260658 | -0.403627613 |
| Hypertension | 0.400975479 | 0.530570972 |
| Diabetes mellitus | 0.706919728 | 0.516704426 |
| Posterior location | 0.222613974 | 0.269602037 |
| Maximum diameter | 0.021824141 | 0.064137973 |
| Neck width | 0.010165054 | -0.038222637 |
| Multiple aneurysms | -0.063635222 | 0.510811101 |

Note: The clinical model contains the eight baseline covariates. The combined model additionally contains AEPCS. Predicted probability is calculated as:

$$p=\frac{exp(\eta)}{1+exp(\eta)}$$

$$\eta=\beta_{0}+\sum_{j}\beta_{j}X_{j}$$

For each model, $\eta$ is the linear predictor, $\beta_{0}$ is the fitted intercept, and $\beta_{j}$ and $X_{j}$ are the coefficient and value of predictor $j$, respectively. The clinical model excludes the AEPCS term.

Age is in years; diameter and neck width are in millimeters. Binary coding: male=1, hypertension present=1, diabetes present=1, posterior location=1, and multiple aneurysms=1; reference categories are female, absent histories, anterior location, and a single aneurysm. AEPCS uses the fixed development scale. These coefficients define the apparent fitted models; optimism-corrected calibration metrics are not substituted for the fitted regression coefficients.

Supplementary Table 8. Sensitivity of spline results to knot specification

| Knots | Percentiles | Knot values | Overall LRT df | Overall P | Nonlinearity LRT df | Nonlinearity P |
| --- | --- | --- | --- | --- | --- | --- |
| 3 | 10%,  50%,  90% | −1.236764,  −0.063781,  1.355783 | 2 | <0.001 | 1 | 0.802 |
| 4 | 5%,  35%,  65%,  95% | −1.537672,  −0.452228,  0.329985,  1.601709 | 3 | <0.001 | 2 | 0.047 |

Note: Models adjusted for the same eight covariates and used all 484 patients. Nonlinearity tests compare each spline model with the linear-score model; overall tests compare it with the covariate-only model. These exploratory results indicate sensitivity to knot specification and do not establish that either an exactly linear or a particular nonlinear form is uniquely correct. LRT, likelihood-ratio test; df, degrees of freedom.

Supplementary Table 9. Apparent and optimism-corrected performance of the multivariable model with 95% confidence intervals

| Performance measure | Apparent estimate | Apparent 95% CI | Optimism-corrected estimate | Optimism-corrected 95% CI |
| --- | --- | --- | --- | --- |
| AUC | 0.853 | 0.819–0.903 | 0.834 | 0.795–0.890 |
| Brier score | 0.089 | 0.069–0.103 | 0.096 | 0.075–0.110 |
| Joint calibration intercept | 0.000 | — | −0.115 | −0.178 to −0.086 |
| Calibration slope | 1.000 | — | 0.897 | 0.870–0.913 |

Note: The model included AEPCS and eight prespecified covariates in the primary cohort of 484 patients with 71 outcome events. Corrected point estimates were obtained using the original 2,000-resample full-pipeline bootstrap analysis (seed 20261004). Percentile confidence intervals were calculated using 2,000 ordinary patient-level outer resamples and 2,000 inner resamples per outer sample (seed 20261006; R type-6 quantiles). Score construction and model fitting were repeated within each training sample, and training-derived parameters were retained for evaluation in the corresponding test sample. Calibration intercept and slope were estimated jointly by logistic regression of outcome on the model linear predictor. Apparent values of 0 and 1 are structural values for the model evaluated in its fitting data; their confidence intervals are therefore omitted.

Among 4,000,000 inner samples, 1,028 (0.026%) had an empty outcome cell in a binary-predictor stratum, indicating separation. These samples were retained using the original unpenalized glm.fit settings, with convergence tolerance 10⁻¹⁰ and a maximum of 100 iterations. Thirty-eight fits generated warnings about probabilities numerically equal to 0 or 1. No resamples were excluded. Re-fitting the separated samples at tolerance 10⁻¹² changed the joint-intercept CI lower limit from −0.178 to −0.179; all other corrected confidence limits were unchanged.

Supplementary Figure 1. Participant flow for the primary and expanded sensitivity cohorts.

Of 556 potentially eligible patients screened, 23 underwent intraoperative conversion from intended stent-assisted coil embolization (SAC) to flow-diverter treatment. Among 533 patients who underwent completed SAC, 16 had incomplete analytic data: 3 lacked previous-medical-history information, 5 lacked contrast-volume measurements, and 8 lacked 28-day outcome data. The complete-data SAC cohort comprised 517 patients. Exclusion of 33 patients with definite intraprocedural complications requiring rescue treatment—15 with cerebral vasospasm, 11 with thrombus formation, and 7 with aneurysm rupture with hemorrhage—yielded the 484-patient primary cohort, with 71 composite outcome events. Reinclusion of these 33 patients yielded the 517-patient expanded sensitivity cohort, with 73 composite outcome events. The composite outcome comprised symptomatic cerebral infarction, symptomatic intracranial hemorrhage, or all-cause death within 28 days after SAC. Intraprocedural adverse events were not automatically counted as primary outcomes. Exclusion categories were mutually exclusive in the reported cohort assembly.

Supplementary Figure 2. Restricted cubic spline analysis of the association between AEPCS and major periprocedural complications.

The logistic regression model used three knots at the 10th, 50th, and 90th percentiles of the Aneurysm Endovascular Procedural Complexity Score (AEPCS; −1.237, −0.064, and 1.356, respectively) and adjusted for age, sex, hypertension, diabetes mellitus, posterior-circulation location, maximum aneurysm diameter, neck width, and multiple aneurysms. The solid curve shows adjusted odds ratios relative to AEPCS = 0, and the shaded area represents pointwise 95% Wald confidence intervals. The y-axis uses a logarithmic scale, and dotted vertical lines indicate knot positions. All 484 patients in the primary cohort were included in model fitting; the displayed curve spans the 1st–99th percentiles of AEPCS. Likelihood-ratio tests yielded P < 0.001 for the overall association (2 degrees of freedom) and P = 0.802 for nonlinearity (1 degree of freedom). Four-knot sensitivity results are reported in Supplementary Table 8 and indicate sensitivity to knot specification.
